# Improved prediction of adverse events in hypertrophic cardiomyopathy with quantitative late gadolinium enhancement and myocardial strain

**DOI:** 10.1186/1532-429X-18-S1-P142

**Published:** 2016-01-27

**Authors:** Vikram Brahmanandam, Emily A Ruden, Subha V Raman

**Affiliations:** Internal Medicine, Cardiology, Ohio State University Medical Center, New York, NY USA

## Background

Risk prediction for sudden death remains imperfect in HCM. We compared CMR-derived LV fibrosis and strain measures to outcomes in a cohort of HCM patients.

## Methods

Consecutive patients undergoing CMR on identical 1.5 Tesla scanners to evaluate HCM were identified at a tertiary referral center. LV fibrosis was measured using a previously validated iterative technique (cmr42, Circle Cardiovascular Imaging) from LGE images acquired using standard inversion recovery techniques. LV volume, mass and EF were assessed from contiguous short axis SSFP cines. LV circumferential and radial strain were measured from a single mid-SAX SSFP cine (Trufi Strain, Siemens). Sustained VT/VF, ICD placement, appropriate ICD therapy, CHF admission, septal reduction and/or death comprised the Event Group, n = 13 and those without the No Event Group, n = 34. Baseline characteristics were reported as mean ± SD for continuous variables and frequencies for categorical variables with group differences examined by t and χ2 tests. A Shapiro-Wilk test was used to evaluate normality and a Wilcoxon-Kruskal-Wallis test was used for non normal distributions. Linear regression analysis evaluated the independent association of predictive variables and receiver operator curves were generated (JMP, SAS).

## Results

**Figure 1:**
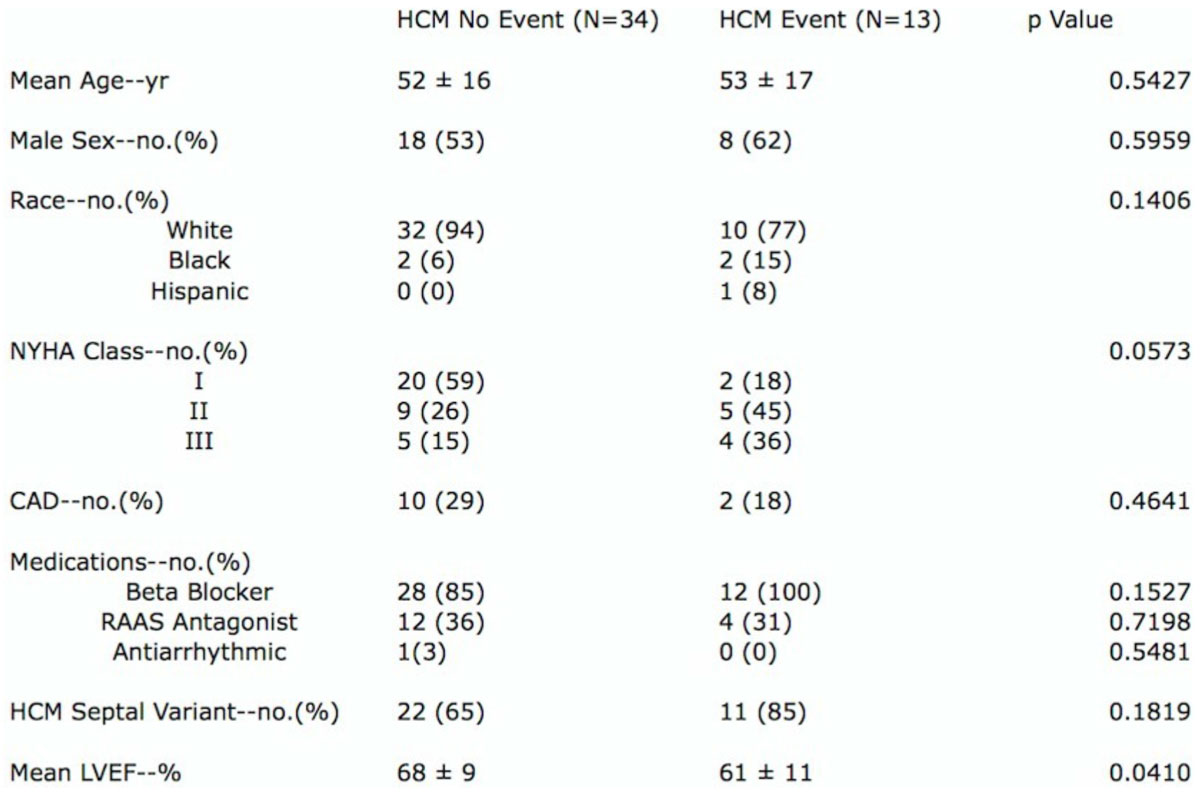
**Baseline Characteristics**.

**Figure 2:**
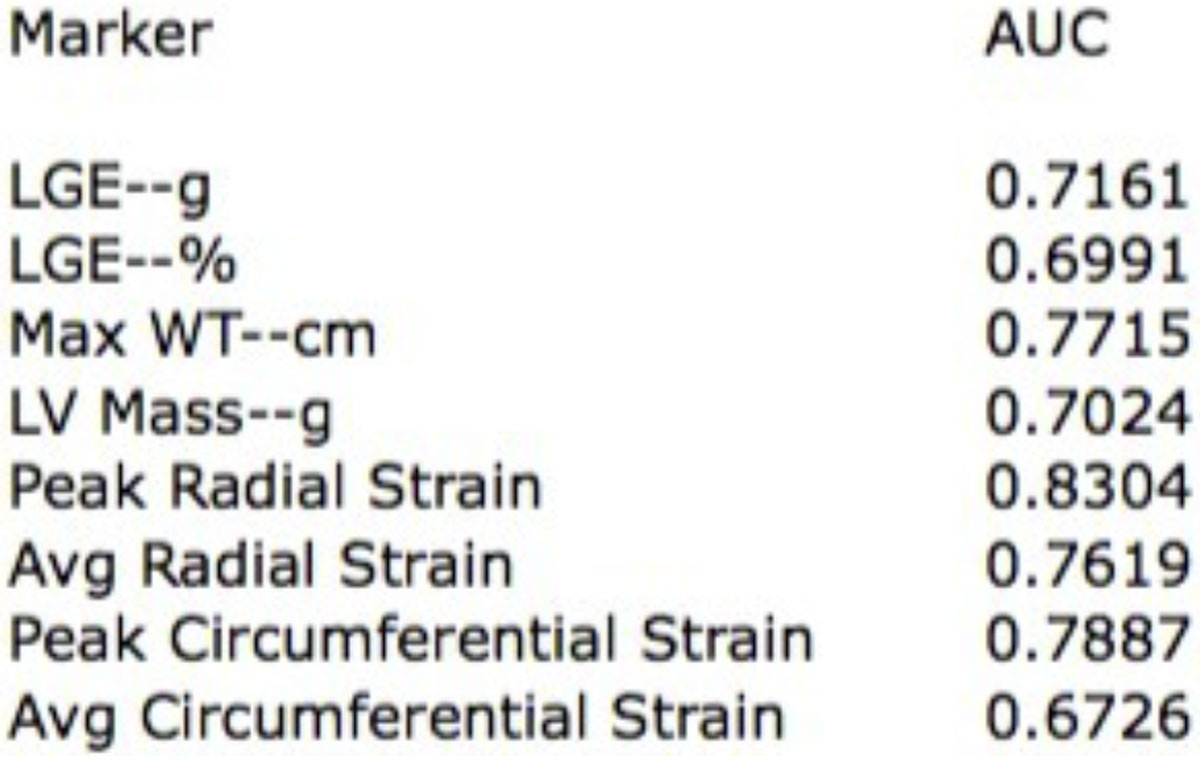
**ROC AUC**.

At a median of 12 months post-CMR, 13 of 47 identified patients experienced an event (Figure 1). While prevalence of traditional risk markers was similar in both groups, myocardial scar burden was significantly greater in those with vs without events (23.0 [10.0-66.5] vs 12.0 [6.0-22.2] g, p = 0.024) as was maximum wall thickness (2.3 [2.1-2.9] vs 2.0 [1.6-2.3] cm, p = 0.004). Peak systolic strain was significantly lower in those with events, both radial (35.4 ± 9.7 vs 51.4 ± 13.2 %, p < 0.001) and circumferential (-14.9 ± 3.5 vs -19.3 ± 4.1 %, p = 0.0024). Peak radial strain, peak circumferential strain and WT had the largest AUCs (0.83, 0.79 and 0.77 respectively (Figure 2).

## Conclusions

LV strain, fibrosis burden and wall thickness contribute to risk stratification in HCM beyond clinical predictors. These findings support further studies to develop multivariable prediction models that incorporate multiple CMR measures.

